# The Combined Inhibition of Autophagy and Diacylglycerol Acyltransferase-Mediated Lipid Droplet Biogenesis Induces Cancer Cell Death during Acute Amino Acid Starvation

**DOI:** 10.3390/cancers15194857

**Published:** 2023-10-05

**Authors:** Maida Jusović, Pia Starič, Eva Jarc Jovičić, Toni Petan

**Affiliations:** 1 Department of Molecular and Biomedical Sciences, Jožef Stefan Institute, SI-1000 Ljubljana, Slovenia; maida.jusovic@gmail.com (M.J.); pia.staric@ijs.si (P.S.); eva.jarc.jovicic@ijs.si (E.J.J.); 2 Jožef Stefan International Postgraduate School, SI-1000 Ljubljana, Slovenia

**Keywords:** lipid droplets, autophagy, nutrient starvation, diacylglycerol acyltransferase, cell death, cancer

## Abstract

**Simple Summary:**

In this study, the relationship between autophagy and lipid droplets in the response of cancer cells to starvation was investigated. Under conditions of amino acid deprivation, autophagy was triggered and led to lipid droplet accumulation through diacylglycerol acyltransferase (DGAT)-mediated neutral lipid synthesis. Combined inhibition of autophagy and lipid droplet biogenesis during acute amino acid starvation was lethal for HeLa cervical cancer cells, but not for MDA-MB-231 breast cancer cells.

**Abstract:**

Lipid droplets (LDs) are dynamic organelles involved in the management of fatty acid trafficking and metabolism. Recent studies suggest that autophagy and LDs serve complementary roles in the protection against nutrient stress, but the autophagy–LD interplay in cancer cells is not well understood. Here, we examined the relationship between autophagy and LDs in starving HeLa cervical cancer- and MDA-MB-231 breast cancer cells. We found that acute amino acid depletion induces autophagy and promotes diacylglycerol acyltransferase 1 (DGAT1)-mediated LD accumulation in HeLa cells. Inhibition of autophagy via late-stage autophagy inhibitors, or by knocking down autophagy-related 5 (ATG5), reduced LD accumulation in amino acid-starved cancer cells, suggesting that autophagy contributes to LD biogenesis. On the contrary, knockdown of adipose triglyceride lipase (ATGL) increased LD accumulation, suggesting that LD breakdown is mediated by lipolysis under these conditions. Concurrent inhibition of autophagy by silencing ATG5 and of LD biogenesis using DGAT inhibitors was effective in killing starving HeLa cells, whereas cell survival was not compromised by suppression of ATGL-mediated lipolysis. Autophagy-dependent LD biogenesis was also observed in the aggressive triple-negative MDA-MB-231 breast cancer cells deprived of amino acids, but these cells were not sensitized to starvation by the combined inhibition of LD biogenesis and autophagy. These findings reveal that while targeting autophagy-driven and DGAT-mediated LD biogenesis reduces the resilience of HeLa cervical cancer cells to amino acid deprivation, this strategy may not be successful in other cancer cell types.

## 1. Introduction

Cancer has been linked to various alterations in lipid metabolism [1,2,3,4]. Cancer cells reprogram their lipid metabolism to ensure energy production and membrane biogenesis and to support signaling pathways that are essential for their rapid growth and proliferation [4]. They can also use alternative and opportunistic modes of nutrient acquisition to survive in the harsh tumor microenvironment characterized by intermittent nutrient and oxygen supply [5]. In one of their major adaptive responses to stress stimuli, cancer cells switch from their metabolic dependency on glycolysis to fatty acid (FA) oxidation [5,6]. However, an elevated flux of FAs is detrimental to cells, as it induces a variety of lipotoxic effects associated with membrane and organelle dysfunction [7,8,9,10]. Lipid droplets (LDs) are emerging as essential organelles for the management of FA lipotoxicity and the control of various aspects of FA metabolism in the cell [11,12,13,14,15]. 

LDs have a unique structure among organelles, with a hydrophobic neutral lipid core covered by a phospholipid monolayer embedded with different proteins [13,16,17]. Within their core, LDs primarily store triacylglycerols (TAGs) and cholesterol esters (CEs). Increased accumulation of LDs has been demonstrated in several malignancies, including breast, brain, and colon cancer [14,18,19]. Interestingly, LD formation is driven by a variety of stressors, which are also characteristic for the tumor microenvironment, including both nutrient excess and deficiency, hypoxia and oxidative stress, as well as autophagy [14,19,20]. In addition to their primary role as energy depots, LDs also serve as buffers that can minimize lipotoxicity [21,22,23,24] and also support mitochondrial metabolism and cell survival during prolonged starvation [9,25,26,27,28,29]. 

Emerging studies point at a complex relationship between autophagy and LD metabolism. Autophagy is a conserved cellular recycling mechanism triggered in response to various stressful conditions, including nutrient excess or deprivation, to deliver excess/damaged cellular material to lysosomes and provide cells with essential nutrients and building blocks [30,31,32]. Autophagy can play a dual role in LD metabolism. Under certain conditions, it may contribute to LD biogenesis [9,28]. On the other hand, autophagy can be involved in the selective degradation of LDs, a process known as lipophagy [33,34]. In addition, LDs have been shown to promote the formation of essential autophagic structures (i.e., elongation of the phagophore membrane and formation of autophagosomes) [35,36,37]. Furthermore, LD–autophagosome/lysosome interactions may serve other functions beyond lipid transfer and energy production, including protein transport [38]. The control of cell homeostasis, cell survival, and cell death are mediated by the complex interplay between lipid metabolism and autophagy. For instance, studies with mouse embryonic fibroblasts (MEFs) have shown that acute amino acid starvation or mammalian target of rapamycin complex 1 (mTORC1) inhibition activates autophagy, which provides FAs that drive LD biogenesis [9,28]. In this instance, LD biogenesis depends on diacylglycerol acyltransferase 1 (DGAT1) and occurs to protect cells from mitochondrial damage caused by an overload of autophagy-derived FAs and their conversion into acylcarnitines [9]. LDs function as a sophisticated buffering system under these conditions as they take up autophagy-derived FAs but also fine-tune their release via ATGL-mediated lipolysis, but not lipophagy, to support mitochondrial adenosine triphosphate (ATP) production. It is not clear whether a similar interplay between LDs and autophagy is employed by different types of cancer cells for survival during various conditions of nutrient deficiency.

In this study, we investigated the associations between autophagy and LD metabolism in starving HeLa cervical- and MDA-MB-231 breast cancer cells with the aim to target this relationship and compromise cancer cell survival during acute nutrient stress. We chose these two cell lines based on their distinct LD metabolic properties revealed in our previous work [23,39,40]. They display significant differences in LD content and turnover upon exposure to various LD-inducing agents and in response to cultivation at various cell densities. Here, we demonstrate that the survival of HeLa cervical cancer cells during amino acid starvation depends on DGAT-mediated LD biogenesis and autophagy. We found that the inhibition of autophagy compromised LD formation and promoted HeLa cancer cell death. Autophagy-driven LD biogenesis also occurred in amino acid-starved MDA-MB-231 breast cancer cells, but inhibition of autophagy and LD biogenesis was not lethal for these cells.

## 2. Materials and Methods

### 2.1. Materials

HeLa human cervical adenocarcinoma, triple negative breast cancer cells (MDA-MB-231), and RPMI-1640 medium were obtained from ATCC (Manassas, VA, USA). Dulbecco’s modified Eagle’s medium with high glucose and GlutaMAX supplement (DMEM-GlutaMax), heat inactivated fetal bovine serum (FBS), and Dulbecco’s phosphate-buffered saline (DPBS) were from Gibco (Billings, MT, USA), and TrypLE Select and Opti-MEM from Life Technologies (Carlsbad, CA, USA). BODIPY 493/503 (cat. no. D3922), BODIPY FL C12 (cat. no. D3822), BODIPY 558/568 C12 (cat. no. D3835), and Lipofectamine RNAiMAX were from Thermo Fisher Scientific (Waltham, MA, USA) and Hoechst 33342 nuclear stain, Cyto-ID autophagy detection kit 2.0, and chloroquine (CQ) from Enzo Life Sciences (Farmingdale, NY, USA). Human ATGL- and ATG5-targeting siRNAs and AllStars Negative Control siRNA were from Qiagen (Hilden, Germany). Autophagy inhibitors bafilomycin A1 (BafA1), rapamycin (Rap), and 3-methyladenine (3-MA) were from Cayman (Ann Arbor, MI, USA), T863 (DGAT1 inhibitor), PF-06424439 (DGAT2 inhibitor), essentially fatty acid-free (EFAF) bovine serum albumin (BSA) (cat. no. A7511), and Nile Red from Sigma-Aldrich (St. Louis, MO, USA). β-actin antibodies (cat. no. NB600-532) were purchased from Novus Biologicals (Cambridge, UK), LC3 (cat. no. #2775), ATG5 (cat. no. #12994), and ATGL (cat. no. #2138) antibodies from Cell Signaling (Danvers, MA, USA), and horseradish-peroxidase-labelled secondary antibodies from Jackson ImmunoResearch Laboratories (West Grove, PA, USA). For Western Blot analysis, we used WB-grade BSA (#A7030, Sigma-Aldrich, St. Louis, MO, USA). All other chemicals were of at least analytical grade and purchased from Sigma-Aldrich (St. Louis, MO, USA) or Serva (Heidelberg, Germany).

### 2.2. Cell Culture and Treatments

HeLa cells were cultured in DMEM-GlutaMAX and MDA-MB-231 cells in RPMI-1640 supplemented with 10% FBS (complete media). Unless otherwise indicated, HeLa- and MDA-MB-231 cells were seeded in 24-well plates at a concentration of 3 × 10^4^ cells/well and 6 × 10^4^ cells/well, respectively. Depending on the experimental goal, two main procedures of acute cell starvation were performed following cell seeding and attachment for 24 h in complete medium, including (a) serum-free mild starvation in DMEM-GlutaMax/RPMI-1640 and (b) severe, amino acid and serum starvation in HBSS medium, both supplemented with 0.02% EFAF-BSA. In case of low glucose starvation, cells were starved in glucose- and glutamine-depleted RPMI-1640 medium (Biological Industries, Cromwell, CT, USA) supplemented with 1 g/L glucose and 2 mM L-glutamine. In experiments with pre-starvation, cells were pre-starved in RPMI-1640 medium containing 0.02% BSA for 24 h. The pre-starvation medium was then replaced with either complete medium, serum-free medium, HBSS, or low-glucose medium. DGAT and autophagy inhibitors were added to culture media during the acute starvation phase.

### 2.3. Gene Silencing Using Small Interfering RNA (siRNA)

In a 24-well plate, reverse transfection was performed on cancer cells at a concentration of 3 × 10^4^ cells/well (HeLa) or 6 × 10^4^ cells/well (MDA-MB-231), whereas for 6-well plates (primarily used for seeding cells for Western Blot analysis), cell density was 1.5 × 10^5^ cells/well (HeLa) or 3 × 10^5^ cells/well (MDA-MB-231). Transfection was performed with 20 nM (mixture of two siRNAs for single-gene silencing) or 40 nM (mixture of four siRNAs for double-gene silencing) total siRNA targeted at ATGL and/or ATG5, or with 20 nM or 40 nM AllStars Negative Control siRNA (Qiagen, Hilden, Germany), used as negative control (SCR) in all experiments. Transfection complexes were generated using 1 μL/well in 24-well plates and 7.5 uL/well in 6-well plates of lipofectamine RNAiMAX in Opti-MEM medium in accordance with guidelines of manufacturer.

### 2.4. Flow Cytometry Analysis of Cellular LD Content

LD analysis was performed as described previously [27]. Briefly, cells were seeded in complete medium in 24-well culture plates at concentration of 3 × 10^4^ cells/well (HeLa) or 6 × 10^4^ cells/well (MDA-MB-231), left to adhere for 24 h, and then treated with a mixture of 20 μM T863 and 20 μM PF-06424439 (DGATi) inhibitors for blocking LD formation, or with 10 mM 3-MA, 1 nM (MDA-MB-231) or 5 nM (HeLa) BafA1, 50 μM (MDA-MB-231), or 100 μM CQ for inhibition of autophagy for 4, 6, 16, or 24 h in serum-free or amino acid-free media. After the cells were collected, the pellet was resuspended in 500 μL of 1 μg/mL Nile Red solution in DPBS and incubated in the dark for 10 min before flow cytometry measurements were taken using a BD FACSCalibur system (BD Biosciences, Franklin Lakes, NJ, USA) equipped with a 488 nm Ar-ion laser, and CellQuest software 6.0 (BD Biosciences, Franklin Lakes, NJ, USA). The FL1 filter was used to capture fluorescence signals from at least 2 × 10^4^ events per sample.

### 2.5. Cyto-ID Autophagy Detection Flow Cytometry Assay

MDA-MB-231 cells were seeded in complete medium in 24-well culture plates at concentration of 6 × 10^4^ cells/well. After 24 h, the cells were washed and treated with 1 nM BafA1 and/or 10 mM 3-MA in serum-free RPMI 1640 or HBSS medium containing 0.02% FAF-BSA for the next 24 h. For analysis, the cells were washed with HBSS containing 0.02% FAF-BSA and detached. After centrifugation at 1000 rpm for 5 min, the pellet was resuspended with 500 μL of phenol-free RPMI-1640 or HBSS medium. After second centrifugation, the pellet was resuspended in the Cyto-ID Green staining solution prepared in phenol-free RPMI-1640 or HBSS medium according to manufacturer’s instructions. Cells were incubated at 37 °C and 5% CO_2_ for 30 min. After the incubation, the cells were washed in phenol-free RPMI-1640 or HBSS medium and collected for analysis using a BD FACSCalibur system (BD Biosciences, Franklin Lakes, NJ, USA). The FL-1 signal of Cyto-ID was captured on at least 1.2 × 10^4^ events per sample.

### 2.6. LC3 Immunostaining

HeLa cells were seeded on glass coverslips in complete medium at a density of 3 × 10^4^ cells/coverslip. After 24 h, the cells were washed and treated with 100 µM CQ in serum-free DMEM Glutamax or HBSS for 16 h. HeLa cells reverse transfected with ATG5 siRNA or control siRNA were subjected to nutrient deprivation in HBSS without additional treatment with autophagy inhibitors. After 16 h starvation, the cells were washed with DPBS, fixed in 4% paraformaldehyde in DPBS for 15 min at room temperature, and permeabilized with 0.1% Triton X-100 in DPBS (DPBST) for 5 min. Fixed cells were blocked with 3% BSA in DPBST for 1 h at room temperature and incubated overnight at 4 °C with an anti-LC3 primary antibody (Cell Signaling, Danvers, MA, USA, cat. no. #2775) diluted at a ratio of 1:200. After washing with DPBS, a secondary antibody conjugated with Alexa Fluor 488 (1:1000 dilution; Thermo Fisher, Waltham, MA, USA, cat. no. #A11070) was added to the cells for 1 h at room temperature in the dark. LDs were stained with the Lipi-Deep Red dye diluted at 1:1000 in DPBS (Dojindo, Kumamoto, Japan, cat. no. #LD04-10) for 30 min at room temperature in the dark. Cells were then washed with DPBS and mounted on slides using mounting medium with DAPI (Thermo Fisher, Waltham, MA, USA, cat. no. #P36962). Images were captured using a confocal laser scanning microscope (LSM 710; Carl Zeiss, Jena, Germany) and processed using the Zen software 8.0 (Carl Zeiss, Jena, Germany).

### 2.7. Live Cell Imaging of LDs

The BODIPY 493/503 neutral lipid dye was used to visualize LDs via live-cell confocal imaging. For this purpose, cells were first washed twice with DPBS and then stained with staining mixture containing 1 μg/mL BODIPY 493/503 and 1 μg/mL Hoechst dye in culture medium without serum depending on the cell type and starvation condition for 15 min in a CO_2_ incubator. Mitochondria were visualized by staining cells with 100 nM Mitotracker Red FM (Invitrogen, Waltham, MA, USA) in serum-free culture medium for 30 min in the cell culture incubator. After the staining, the cells were washed twice with DPBS and fresh culture medium that had been used prior to the staining added to the cells. Live cell imaging was performed using an inverted epifluorescence (Zeiss Axio Observer Z1; Carl Zeiss, Jena, Germany) or confocal laser scanning microscope (LSM 710; Carl Zeiss, Jena, Germany) depending on the selected time points in starvation conditions. Images were processed using the Zen software 8.0 (Carl Zeiss, Jena, Germany).

### 2.8. Live Cell Imaging of Autophagosomes Stained with Cyto-ID

Autophagosomes were visualized using the Cyto-ID autophagy detection kit. For imaging, the cells were washed twice using cultivating medium without phenol red or HBSS and stained with microscopy dual detection solution containing Cyto-ID and Hoechst dyes dissolved in phenol-red-free medium or HBSS according to manufacturer’s instructions. Cells were stained for 30 min at 37 °C and 5% CO_2_ and then washed twice with phenol-red-free or HBSS medium and left in fresh phenol-red-free or HBSS cultivating medium containing 0.02% FAF-BSA. Live cell imaging was performed using confocal microscopy (Carl Zeiss, Jena, Germany).

### 2.9. Pulse-Chase Imaging Experiments for Detection of LD Formation

LD dynamics in HeLa cells exposed to feeding (serum-rich) and starvation conditions (serum-free and amino acid-free) was determined with pulse-chase experiments using exogenously added fluorescent FA analogues BODIPY FL C12 and BODIPY 558/568 C12. The FA analogues were complexed to 0.02% EFAF-BSA in serum-depleted culture medium (for mild starvation) or HBSS (for severe starvation) for 1 h at room temperature before its addition to the cell culture. Cells were first reverse transfected with ATG5-coding siRNA or SCR control and left in complete medium for 24 h. After the feeding, the cells were washed and medium replaced with serum-free medium containing 1 µM BODIPY 558/568 C12 and a mixture of DGAT inhibitors (pre-starvation phase). After 24 h, images of serum-free starved cells were captured using confocal microscopy at zero timepoint (0 h), while remaining cells were washed twice with DPBS and starved for additional 6 h in HBSS before images were taken. Finally, 1 µM of BODIPY FL C12 was added to the cells 15 min prior microcopy.

### 2.10. LD Counting and Diameter Analysis

Live-cell microscopy images of BODIPY 493/503-stained LDs were used for computer image analysis using ImageJ software 1.53t (National Institutes of Health, Bethesda, MD, USA) and the LipidDroplet Counter Plugin as described previously [40]. The analysis was carried out on 32-bit two-dimensional pictures, where the quantities of LDs as well as their sizes were calculated in accordance with the instructions provided by the plugin. The surface areas of the LDs were used to calculate the diameters of LDs. Each sample was subjected to a minimum of 30 cells for analyses.

### 2.11. Western Blot Analysis

Following the steps of serum-free or amino acid-free starvation outlined above, Hela cells were first seeded in complete media in 6-well plates at a density of 1.5 × 10^5^ cells/well and if necessary, reverse transfected with siRNA. Cell lysates were prepared by washing adherent cells in ice-cold DPBS and scraping the cells in Tris-Glycine sodium dodecyl sulfate (SDS) Sample Buffer (2X) (Novex by Life Technologies, Carlsbad, CA, USA) with 800 mM DTT (Sigma-Aldrich, St. Louis, MO, USA) and by adding Halt Protease Inhibitor Cocktail (Thermo Scientific, Waltham, MA, USA) to the mixture. After a ten-minute incubation at 95 °C, the lysates were kept on ice or frozen at –80 °C until Western blot analysis. The Pierce 660 nm Protein Assay (Thermo Scientific, USA) was used to determine the total protein concentration. Then, 5 μg of total protein was separated on 12.5% SDS-PAGE gel for LC3 and 10 μg of total protein on 10% SDS-PAGE gel for ATG5 or ATGL protein detection, and then transferred to polyvinylidene difluoride (PVDF) (Merck Millipore Ltd., Darmstadt, Germany) or nitrocellulose membrane, respectively (Serva, Heidelberg, Germany). Following transfer, membranes were blocked for each protein separately, including 1 h in 5% non-fat dry milk in TBS/0.1% Tween-20 (TBST) (ATGL), 3% BSA (#A7030) in TBST for LC3, and 5% BSA in TBST for ATG5, or in 1% Western blocking reagent (WBR) (Roche Applied Science, Penzberg, Germany) in TBS for β-actin. After blocking, membranes were incubated overnight at 4 °C in the presence of anti-rabbit primary antibodies at 1:1000 (ATGL, ATG5, and LC3) or 1:5000 (β-actin) dilution. The primary antibodies were diluted as follows: ATGL in 5% non-fat dry milk in TBST, ATG5 and LC3 in 5% BSA in TBST, and β-actin in 0.5% WBR in TBS. Following multiple washing steps in TBST, the membranes were subjected to 60 min incubation with HRP-conjugated secondary antibodies (1:10,000) diluted in the primary antibody solution. Lumi-Light Western Blotting Substrate (Roche Applied Science, Penzberg, Germany) was used for visualization and imaging performed on Gel Doc XR system (Bio-Rad, Hercules, CA, USA).

### 2.12. TMRM/YO-PRO-1 Cell Death Assay

Cell death was determined using TMRM/YO-PRO-1 assay and flow cytometry as described previously [23]. Briefly, cells were seeded in complete medium in 24-well plates at concentration of 3 × 10^4^ cells/well and allowed to adhere for 24 h. After attachment, cells were treated with different inhibitors, blocking LD formation (40 μM mixture of DGATi) or autophagy (5 nM bafilomycin A1), for the time indicated in experimental settings. For analysis, cells were harvested and the pellet re-suspended in 100 μL of 25 nM TMRM in DPBS and incubated in the dark for 15 min. To detect dead cells, YO-PRO-1 was added to a final concentration of 50 nM and cells incubated for additional 10 min. The cell suspension was diluted with 200 μL (D)PBS containing 0.1% FA-free BSA (cat. no. A8806) from Sigma (St. Louis, MO, USA). Flow cytometry using the FL1 and FL3 filters was used to analyze the TMRM and YO-PRO-1 signals from at least 20,000 cells in each sample, respectively. TMRM-negative and YO-PRO-1-positive cells were considered apoptotic.

### 2.13. Statistical Analyses

All statistical analyses were carried out using Prism 9.4.1 (GraphPad Software, Boston, MA, USA). Data are presented as means ± SEM. Statistical significance was determined by one-way or two-way ANOVA, followed by Šidak or Tukey’s post hoc tests. *p* values lower than 0.05 were considered statistically significant.

## 3. Results

### 3.1. Acute Amino Acid Depletion Increases DGAT1-Dependent LD Accumulation in HeLa Cells

**Figure 1:**
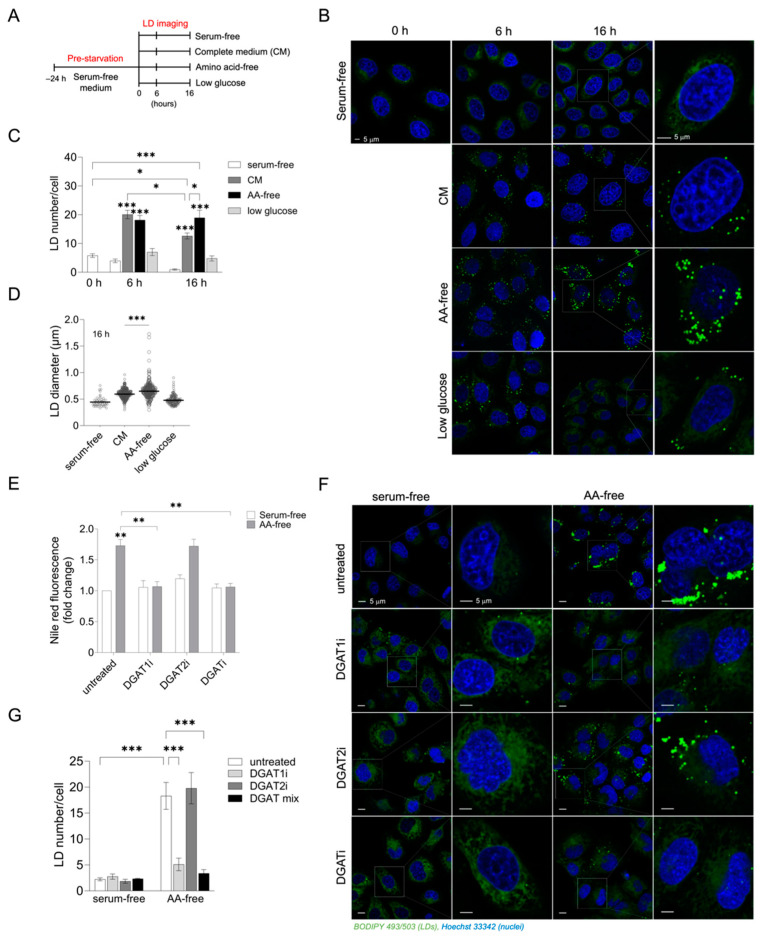
DGAT1 promotes LD biogenesis in response to amino acid withdrawal in HeLa cells. (**A**–**C**) LD levels in HeLa cells pre-starved in serum-free medium (0 h) followed by 6–16 h of growth in different media (complete, serum-free, low glucose, or amino acid (AA)-free media), as shown in the experimental setup scheme (**A**). (**D**) Changes in LD diameters in HeLa cells grown as shown in (**A**). (**E**,**F**) Neutral lipid content and LD abundance in AA-starved HeLa cells treated with DGAT inhibitors. Starved HeLa cells were treated with 20 μM T863 (DGAT1i), 20 μM PF-06424439 (DGAT2i), or both (DGATi). After 16 h of starvation, neutral lipid content and LD abundance levels were determined. (**G**) Changes in LD abundance in serum-starved and AA-starved HeLa cells treated with DGAT inhibitors. (**B**,**F**) Cells were stained with 1 µg/mL BODIPY 493/503 to visualize LDs (green) and Hoechst stain solution to visualize nuclei (blue), and images were analyzed using ImageJ and the LD Counter Plugin (**C**,**D**,**G**). (**E**) Neutral lipid content was measured via Nile red staining and flow cytometry. Data presented are means ± SEM (**C**,**E**,**G**) or geometric means ± SEM (**D**) of at least two independent experiments. Quantification of LD number/cell (**C**,**G**) and size (**D**) was determined from more than 50 (**C**,**D**) or 30 cells/sample (**G**). Results that are statistically significant are indicated (*, *p* < 0.05; **, *p* < 0.01; ***, *p* < 0.001 (one-way ANOVA with Šidak adjustment (**C**,**D**), two-way ANOVA with Tukey adjustment (**E**,**G**))).

Increased LD accumulation has been observed in different human cell lines grown in amino acid-depleted medium [9,28]. To better understand how LD metabolism is regulated in HeLa cervical cancer cells during acute nutrient stress, we first examined changes in the abundance of LDs in response to feeding and different starvation conditions (Figure 1A). In this experiment, HeLa cells were first pre-starved for 24 h in serum-free medium in order to lower initial LD levels. HeLa cells were indeed almost fully depleted of LDs during this pre-starvation phase and there were no further changes in LD abundance in the next 16 h of serum-free cultivation (Figure 1B,C). When cells were switched from a serum-free medium to complete medium, or exposed to starvation in the complete absence of amino acids in HBSS, there was a prominent increase in LD abundance (Figure 1B,C). LD accumulation in amino acid-starved cells reached similar levels to those in fed cells after 6 h of starvation (Figure 1C). However, LD numbers and LD diameters were higher in amino acid-starved cells in comparison with those in fed cells after 16 h of starvation (Figure 1C,D). Cells grown in the low-glucose-containing medium also accumulated LDs, but to a much lesser extent than those cultivated in HBBS or complete media (Figure 1B,C). In addition, when the pre-starvation phase was omitted and HeLa cells were switched directly from the complete medium to either a fresh complete medium or HBSS (Supplementary Figure S1A,B), a significant increase in LD accumulation in comparison with initial LD levels was observed after 16 h of amino acid starvation. On the contrary, LD abundance in fed cells decreased over time relative to initial levels. Thus, with or without pre-starvation-induced LD depletion, HeLa cells accumulate more LDs during starvation in HBSS than when grown in complete media. These results suggest that various conditions of acute starvation have distinct effects on the LD metabolism in HeLa cells, including on LD breakdown due to serum depletion and prominent LD accumulation stimulated by amino acid depletion.

To find out if the observed LD accumulation in amino acid-free conditions is a consequence of de novo TAG synthesis and LD biogenesis, we treated cells with T863 and PF-06424439, inhibitors of DGAT1 and DGAT2 enzymes, respectively [41,42]. We found that inhibition of DGAT1 or a combined inhibition of DGAT1 and DGAT2 enzymes blocked amino-acid-starvation-induced LD accumulation, whereas DGAT2 inhibition alone did not affect the LD number or neutral lipid levels (Figure 1E–G). These results suggest that amino-acid-starvation-induced LD biogenesis in HeLa cells depends on DGAT1 activity.

### 3.2. Inhibition of Autophagy Suppresses Amino-Acid-Starvation-Induced LD Biogenesis in Cancer Cells

**Figure 2:**
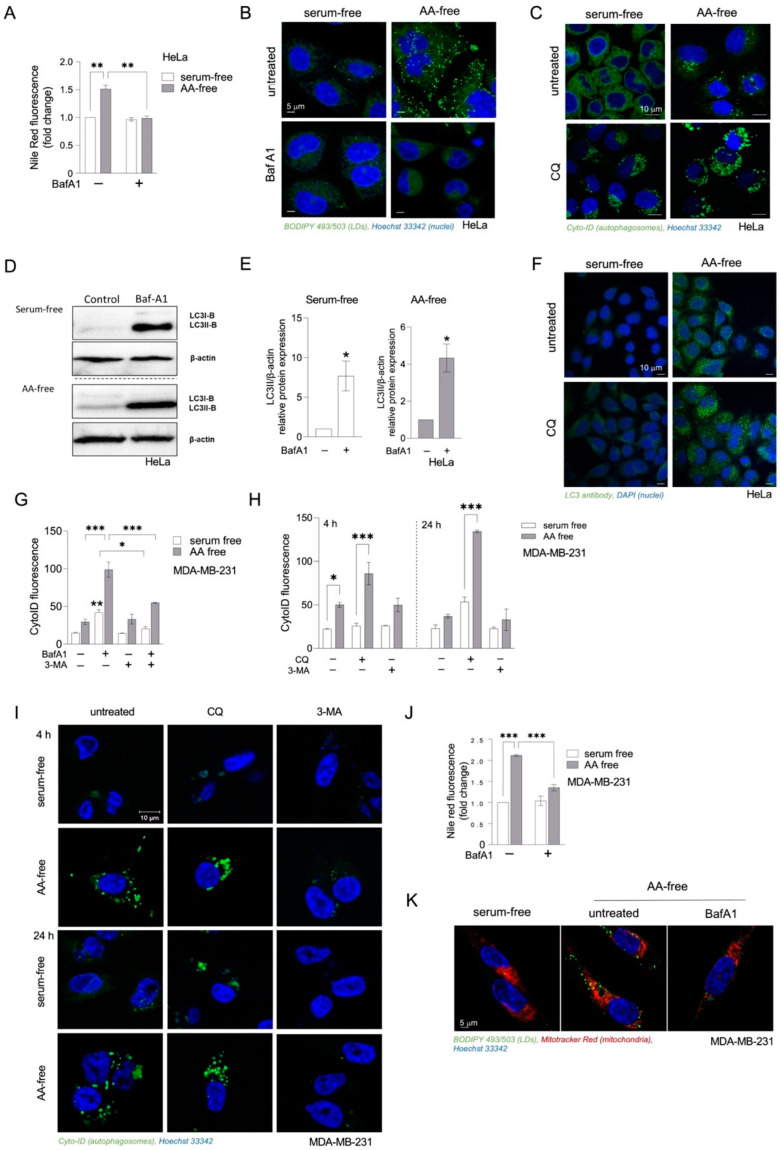
Starvation-induced autophagy contributes to LD accumulation in acutely starved HeLa- and MDA-MB-231 cells. (**A**,**B**) Neutral lipid content in amino acid (AA)- and serum-starved HeLa cells treated with bafilomycin A1 (BafA1). (**C**) Cyto-ID-stained autophagosomes in starving HeLa cells treated with chloroquine (CQ). (**D**,**E**) Autophagic flux in starving HeLa cells estimated by LC3-II turnover in the presence and absence of BafA1. (**F**) LC3 puncta in starving HeLa cells treated with CQ. (**G**–**I**) Changes in autophagic flux in starving MDA-MB-231 cells estimated by Cyto-ID staining of autophagosomal structures in the presence or absence of BafA1, CQ, and 3-methyladenine (3-MA). (**J**) Neutral lipid content in starved MDA-MB-231 cells treated with BafA1. (**K**) Microscopic examination of changes in LD abundance in starving MDA-MB-231 cells in the presence and absence of BafA1. (**A**–**K**) Cancer cells were seeded in complete medium and then starved in serum-free or AA-free media for 16 h (HeLa; **A**–**F**) or 4 h (**H**,**I**) and for 24 h (MDA-MB-231; **G**–**K**). During starvation, the cells were treated with autophagy inhibitors as follows: 1 nM (MDA-MB-231) or 5 nM BafA1 (HeLa), 50 μM (MDA-MB-231) or 100 μM (HeLa) CQ, and 10 mM 3-MA. (**A**,**J**) Neutral lipid content was measured using Nile Red staining and flow cytometry. Data are means ± SEM of two (**A**) or three (**J**) independent experiments. (**B**,**K**) The cells were stained with 1 µg/mL BODIPY 493/503 to visualize LDs (green), Hoechst stain solution to visualize nuclei (blue), and (**K**) with Mitotracker Red to visualize mitochondria (red). The images shown are representative of at least two independent experiments. (**C**,**G**–**I**) Cells were stained with the Cyto-ID dye to visualize (by confocal microscopy) (**C**,**I**) and quantify (by flow cytometry) (**G**,**H**) autophagosomal structures according to manufacturer’s instructions. The images shown and presented data of means ± SEM are representative of two (**C**,**I**) or three (**G**,**H**) independent experiments. (**D**) Cell lysates were analyzed for the presence of basal LC3 (LC3-I) and lipidated form levels (LC3-II) and β-actin control via Western blotting. (**E**) LC3-II protein levels were quantified via densitometry. (**D**,**E**) The blots and data ± SEM shown are representative of two independent experiments. (**F**) Fixed cells were analyzed for the presence of LC3 puncta using immunofluorescence. Nuclei were visualized using DAPI stain. The images shown are representative of one experiment. Results that are statistically significant are indicated (*, *p* < 0.05; **, *p* < 0.01; ***, *p* < 0.001 (two-way ANOVA with Tukey (**A**,**J**) or Šidák (**G**,**H**) adjustment; unpaired *t*-test (**E**)).

In amino acid-starved MEFs, autophagy is strongly induced and mediates the recycling of FAs from membrane phospholipids, which promotes FA storage in TAGs stored within LDs [9,28]. To determine whether autophagy is activated and has an impact on LD abundance in HeLa cells during severe amino acid deficiency, we blocked autophagy by using the late-stage autophagy inhibitors BafA1 or CQ, which suppress lysosomal acidification, and examined their effects on LD accumulation during amino acid starvation. Treatments with BafA1 resulted in suppression of amino-acid-starvation-induced accumulation of neutral lipids (Figure 2A), thereby depleting HeLa cells of LDs (Figure 2B). To confirm the involvement of autophagy under these conditions, we determined changes in autophagosome abundance and autophagic flux. Amino acid starvation caused an increase in autophagosome staining (Figure 2C), LC3-II expression (Figure 2D,E), and LC3 puncta (Figure 2F), indicative of increased autophagosome formation. This increased formation was coupled with increased autophagosome turnover as evidenced by even higher numbers of autophagosomes (Figure 2C), an increased LC3-II expression (Figure 2D,E), and higher LC3 puncta (Figure 2F) when the cells were treated with late-stage autophagy inhibitors CQ and BafA1. To expand the scope of our findings on HeLa cells and to determine if this phenomenon is conserved across other cancer types, we next examined whether autophagy-induced LD accumulation also occurs in aggressive, Ras-driven MDA-MB-231 breast cancer cells. Treating amino acid-starving MDA-MB-231 cells with BafA1 or CQ suppressed autophagic flux resulting in autophagosome accumulation, which was prevented by the autophagy inhibitor 3-MA (Figure 2G–I). BafA1 also reduced amino-acid-starvation-induced neutral lipid levels (Figure 2J) and LD accumulation (Figure 2K), suggesting that autophagy drives LD biogenesis in starving MDA-MB-231 cells.

**Figure 3:**
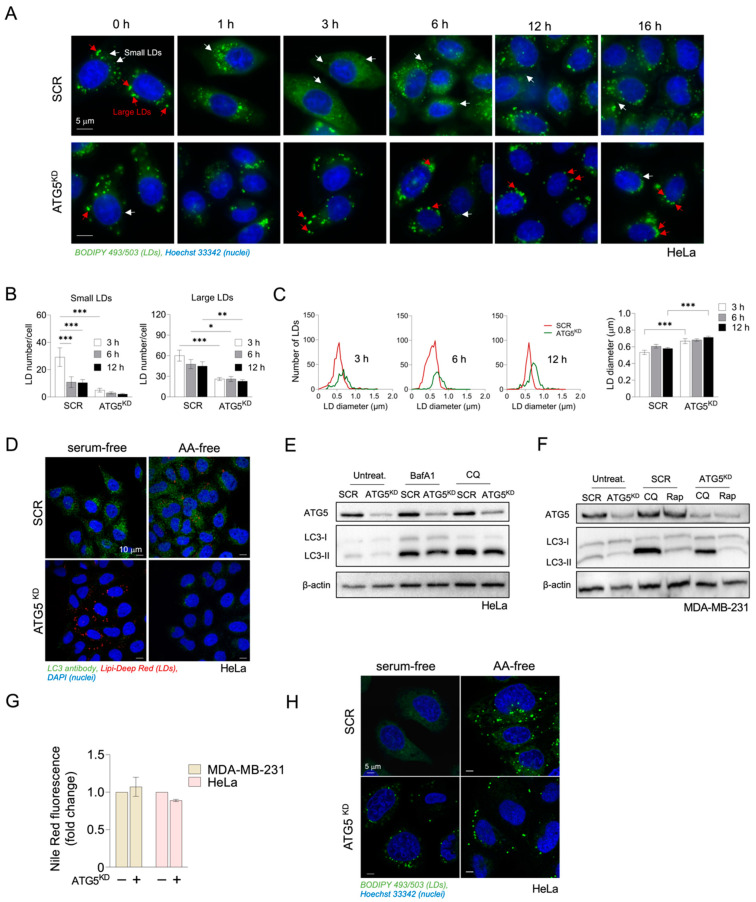
Inhibition of autophagy through ATG5 depletion impairs amino-acid-starvation-induced LD accumulation. (**A**) Microscopic images showing LD abundance in control and ATG5-deficient HeLa cells during starvation in amino acid (AA)-free medium. (**B**,**C**) Changes in the number of small LDs (≤0.41 μm) and large LDs/cell (>0.41 μm) and in the LD diameter in control and ATG5-deficient HeLa cells at 3, 6, and 12 h of starvation in AA-free medium. (**D**) LC3 puncta and LD abundance in control and ATG5-deficient HeLa cells during starvation in AA-free and in serum-free medium. (**E**,**F**) ATG5 protein levels and LC3-II turnover in starving control and ATG5-deficient HeLa (**E**) and MDA-MB-231 (**F**) cells in the presence and absence of BafA1, CQ, or 100 µM Rapamycin (Rap). (**G**,**H**) Cellular neutral lipid content and LD abundance in AA- and serum-starved control and ATG5-deficient HeLa cells. (**A**–**H**) Cancer cells were reverse transfected with 20 nM of ATG5 or non-targeting siRNA and seeded in complete medium following treatment with autophagy inhibitors, as described in Figure 2, in serum-free or AA-free medium for 16 h (HeLa) or 24 h (MDA-MB-231). (**A**,**H**) LDs and nuclei were visualized using 1 µg/mL BODIPY 493/503 and Hoechst stain solution, respectively. The epifluorescence microscopy images from 50 cells/sample of two independent experiments from panel A were analyzed for the number of small LDs and large LDs/cell (**B**) and LD diameter (**C**) at selected time points using ImageJ and the LD Counter Plugin. (**D**) Fixed HeLa cells were analyzed for the presence of LC3 puncta using immunofluorescence. Nuclei and LDs were visualized using DAPI stain and the Lipi-Deep Red dye, respectively. (**E**,**F**) Cell lysates were analyzed for the presence of ATG5 protein level, LC3 basal (LC3-I), and lipidated form levels (LC3-II) and β-actin control via Western blotting. The blots are representative of two independent experiments. (**G**) Neutral lipid levels were quantified using Nile Red dye and flow cytometry. (**B**,**C**,**G**,**H**) Representative images are shown and data presented are means (**B**,**G**) or geometric means (**C**) ± SEM of two (**B**,**C**) or three (**G**,**H**) independent experiments. Results that are statistically significant are indicated (*, *p* <0.05; **, *p* <0.01; ***, *p* <0.001 (one-way ANOVA (**B**,**C**) and two-way ANOVA with Šidák (**G**) adjustment)).

Notably, our time-lapse microscopic examinations revealed the predominant accumulation of many small LDs in HeLa cells after 3 h of amino acid starvation (Figure 3A,B). During longer periods of amino acid starvation, the number of small LDs significantly decreased, whereas the number of large LDs was reduced only minimally (Figure 3B). To confirm that autophagy drives LD biogenesis under these conditions, we silenced ATG5, an essential autophagic gene, which is required for phagophore elongation [43]. In comparison with control cells, the number of small LDs per cell was significantly reduced in ATG5-silenced cells, most evidently at the 3 h time point (Figure 3B). ATG5 depletion reduced the abundance of large LDs as well, but to a lesser extent (Figure 3B). A corresponding increase in average LD diameters was observed in ATG5-deficient cells during starvation (Figure 3C). The decreased presence of small and large LDs in ATG5-silenced cells could be attributed to impaired LD formation resulting from the suppression of autophagy. However, the significant increase in LD diameters in ATG5-depleted cells, as shown in Figure 3C, suggests that autophagy may also play a role in LD breakdown under these conditions. Altogether, these experiments reveal that depleting HeLa cells of ATG5 primarily suppresses amino-acid-starvation-induced LD accumulation.

Furthermore, in ATG5-deficient HeLa cells, we observed a significant decrease in the number of LC3 puncta in both serum-starved and amino acid-starved cells (Figure 3D), which essentially confirmed the desired impairment of autophagosome maturation and blockade of autophagy caused by the silencing of ATG5 (Supplementary Figure S2A). As observed by the reduction in LC3-II protein levels in ATG5-deficient cells compared with cells treated with non-targeting siRNA (SCR), and in the presence of late-stage autophagy inhibitors, ATG5 depletion reduced autophagic flux in both cell lines (Figure 3E,F; Supplementary Figure S2A–F). Interestingly, our flow cytometry measurements did not detect significant changes in bulk neutral lipid levels caused by ATG5 deficiency in amino acid-starved HeLa- and MDA-MB-231 cancer cells (Figure 3G). This is in line with the predominant effect of ATG5 depletion on the abundance of small LDs (Figure 3B,C). However, microscopic examinations comparing the levels of LDs in amino acid- and serum-starved HeLa cells suggested that ATG5 depletion in amino acid-starved cells results in a reduction in LDs to levels comparable to those observed in ATG5-silenced serum-starved cells (Figure 3H). This finding was consistent with the observed reduction in LD abundance induced by BafA1 in amino acid-starved cells (Figure 2A), confirming the role of autophagy in maintaining LD levels during conditions of amino acid starvation. Furthermore, ATG5-deficient serum-starved cells displayed an increase in LD accumulation in comparison with control-serum-starved cells (Figure 3H), indicating a dual role of autophagy in regulating LD turnover that appears to be dependent on the type of starvation.

**Figure 4:**
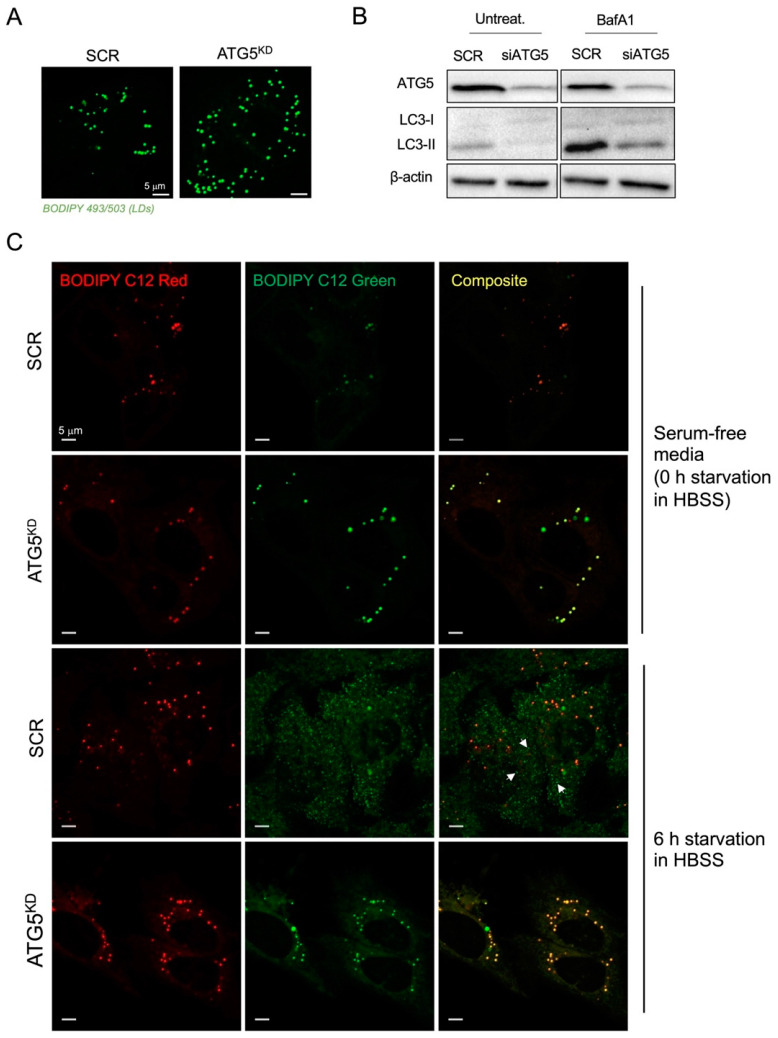
ATG5 contributes to LD breakdown during nutrient sufficiency, but promotes LD formation during amino acid starvation. (**A**,**B**) LD abundance (**A**) and LC3-II turnover (**B**) in control and ATG5-deficient HeLa cells grown in complete media. (**C**) Live cell imaging of pre-existing (BODIPY C12 Red) and newly formed LDs (BODIPY C12 Green) during HBSS starvation of control and ATG5-deficient HeLa cells. (**A**–**C**) HeLa cells were reverse transfected with ATG5 or SCR siRNA in complete medium. Twenty-four hours later, the medium was replaced with fresh complete medium (**A**,**B**) or serum-free medium containing 1 μM BODIPY C12 Red and a mixture of DGAT1 and DGAT2 inhibitors (DGATi; 20 μM each) and cells were left for the next 24 h (**C**). Starved cells were then treated with 1 μM BODIPY C12 Green added directly to the medium 15 min before images were taken (time point 0 h) or washed and starved in amino acid-free medium for 6 h and then treated with 1 μM BODIPY C12 Green as described and analyzed using live-cell confocal microscopy (**C**). (**A**) LDs were visualized using BODIPY 493/503. (**B**) Cell lysates were analyzed via Western blotting for the presence of ATG5 protein, LC3 basal (LC3-I), and lipidated forms of (LC3-II) and β-actin. The images and blots shown are representative of at least two independent experiments.

We reasoned that the reduction in the number of LDs, coupled with the observed increase in LD diameters in ATG5-deficient cells during HBSS starvation (Figure 3B), might result from the impact of ATG5 depletion on pre-existing LDs that originated from cell growth in complete media prior to the onset of starvation. Namely, we observed that ATG5 silencing leads to elevated accumulation of LDs in cancer cells grown in nutrient-rich media in the presence of serum (Figure 4A) and in serum-free media (Figure 3H). Together with the reduced LC3-II turnover determined in ATG5-deficient HeLa cells grown in complete media (Figure 4B; Supplementary Figure S2G,H), these results suggest that ATG5-mediated lipophagy contributes to LD breakdown during both nutrient sufficiency and during serum starvation. To examine the effects of ATG5 silencing on LD abundance specifically during HBSS starvation, we pre-starved HeLa cells in serum-free medium to reduce the number of existing LDs accumulated during growth in complete media and treated the cells with DGAT inhibitors to prevent de novo LD biogenesis during the pre-starvation. This was followed by a 6 h starvation in HBSS in the absence of DGAT inhibitors to allow for the starvation-induced de novo LD biogenesis. Fluorescent BODIPY-labelled FA analogues were used to discern between pre-existing (red) and de novo-formed LDs (green) [44]. Live-cell confocal microscopy images taken at the end of the pre-starvation phase (0 h, upper panel in Figure 4C) compared with images taken after a 6 h starvation in HBSS (6 h, lower panel in Figure 4C) revealed the selective labelling of de novo-formed LDs with the BODIPY-FA Green dye (marked on Figure 4C with white arrowheads), which were absent in ATG5-deficient cells. The overlapping red and green signals observed in ATG5-deficient cells suggest that these cells mainly contain pre-existing LDs formed in complete medium, but lack the amino-acid-starvation-induced LDs. These findings confirmed that ATG5-depleted HeLa cells have a reduced capacity to form new LDs during starvation in an amino acid-free medium. Overall, our results demonstrate that autophagy is activated during severe amino acid deprivation of cancer cells and that it drives the formation of LDs.

### 3.3. Autophagy-Driven and DGAT-Mediated LD Biogenesis Protect HeLa Cells from Cell Death during Severe Amino Acid Starvation

**Figure 5:**
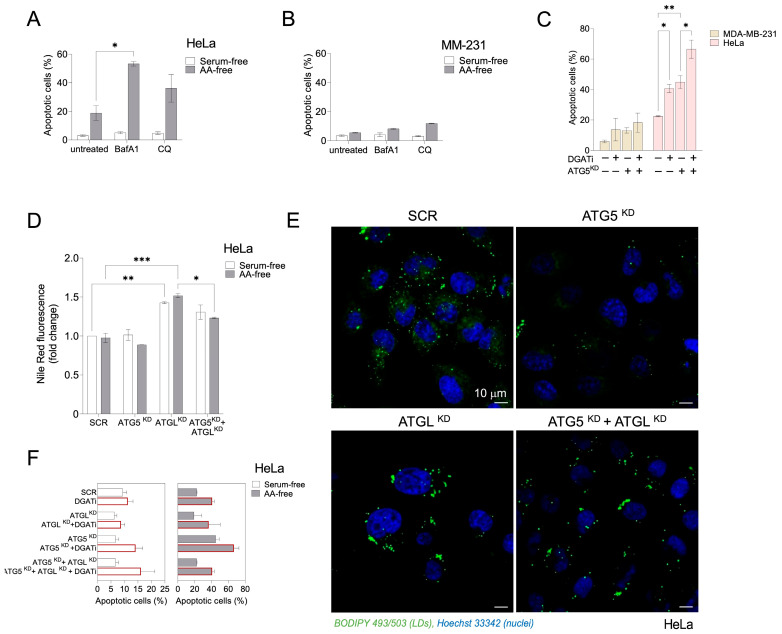
Inhibition of autophagy and DGAT-mediated LD biogenesis increase HeLa cell death, but do not affect MDA-MB-231 cell viability during acute amino acid starvation. (**A**,**B**) Cell death rates in serum- or amino acid (AA)-starved HeLa- (**A**) and MDA-MB-231 (**B**) cells treated with autophagy inhibitors BafA1 and CQ. (**C**) Cell death rates in AA-starved control and ATG5-deficient HeLa- and MDA-MB-231 cells treated with a mix of DGAT1 and DGAT2 inhibitors (DGATi, 20 µM each). (**D**,**E**) Neutral lipid levels (**D**) and LD abundance (**E**) in control, ATG5-deficient, and ATGL-deficient serum- or AA-starved HeLa cells. (**F**) Cell death rates in control, ATG5-deficient, and ATGL-deficient serum- or AA-starved HeLa cells treated with an equimolar mixture of DGAT1 and DGAT2 inhibitors. (**A**–**F**) Cancer cells were seeded in complete medium and then starved in serum-free or AA-free medium for 6 h (HeLa; **D**,**E**), 16 h (HeLa; **C**,**F**), or 24 h (HeLa; **A**, MDA-MB-231; **B**,**C**). In panels C and E, the cells were starved in AA-free medium. (**C**–**F**) ATG5 and ATGL silencing was performed via reverse transfection with 20 nM of ATG5 or ATGL siRNA and control cells were transfected with 20 nM of non-targeting siRNA (SCR). The percentage of dead cells was determined via TMRM/YO-PRO-1 double staining and flow cytometry (**A**–**C**,**F**). Neutral lipid content was measured via Nile Red staining (**D**). Cellular LDs in starved cells were visualized using 1 µg/mL BODIPY 493/503 and nuclei using Hoechst stain solution (**E**). Values on graphs are means ± SEM of at least two (**A**–**D**) or three (**F**) independent experiments and results that are statistically significant are indicated (*, *p* < 0.05; **, *p* < 0.01; ***, *p* < 0.001; two-way ANOVA with Tukey’s adjustment).

Autophagy-driven LD biogenesis has been shown to prevent mitochondrial damage and cell death in starving MEFs by sequestering autophagy-derived FAs into LDs [9]. We next investigated whether interfering with LD turnover combined with the inhibition of autophagy alters the capacity of HeLa- and MDA-MB-231 cancer cells to survive severe nutrient stress caused by amino acid deprivation. Inhibition of autophagy by treating HeLa cells with BafA1 significantly increased cell death during amino acid starvation, but not during mild starvation in serum-free media (Figure 5A). In contrast, cell death of MDA-MB-231 cells was not significantly affected by the inhibition of autophagy during mild or severe nutrient stress conditions (Figure 5B). Importantly, both ATG5 depletion and DGAT inhibition significantly increased starvation-induced HeLa cell death, which was further potentiated by a combined impairment of both LD biogenesis and autophagy (Figure 5C). On the contrary, DGAT inhibition and the suppression of autophagy through ATG5 depletion had only a minor effect on the cell death of amino acid-starved MDA-MB-231 breast cancer cells (Figure 5C). This suggests that targeting of both, TAG synthesis and autophagy, effectively promotes HeLa cancer cell death during severe amino acid starvation, while MDA-MB-231 cells are resistant to the inhibition of both processes in these conditions.

Previous studies on amino acid-starved MEFs have shown that the breakdown of autophagy-derived LDs occurs via ATGL-mediated lipolysis [28]. To find out if amino acid-starved HeLa cells employ ATGL in a similar manner under these conditions, we knocked down ATGL using siRNA and examined the changes in LD abundance and morphology. ATGL depletion led to an increase in LD abundance in acutely starved HeLa cells in HBSS medium (Figure 5D,E). Co-inhibition of ATGL and autophagy by silencing ATG5, as compared with the sole depletion of ATGL, resulted in a significant reduction in neutral lipid levels (Figure 5D) and decreased LD accumulation in amino acid-starved HeLa cells (Figure 5E). This suggests that LDs formed as a result of autophagy are primarily broken down through ATGL-mediated lipolysis, rather than lipophagy, under conditions of amino acid starvation. Finally, we observed that ATGL depletion, whether alone or in combination with DGAT inhibition or with ATG5 knockdown, resulted in slightly lower levels of cell death, particularly during amino acid starvation, but these effects were not statistically significant (Figure 5F). Thus, ATGL contributes to the breakdown of autophagy-derived LDs in amino acid-starved HeLa cells, but inhibiting lipolysis though ATGL depletion does not significantly increase HeLa cell death under these conditions.

## 4. Discussion

**Figure 6:**
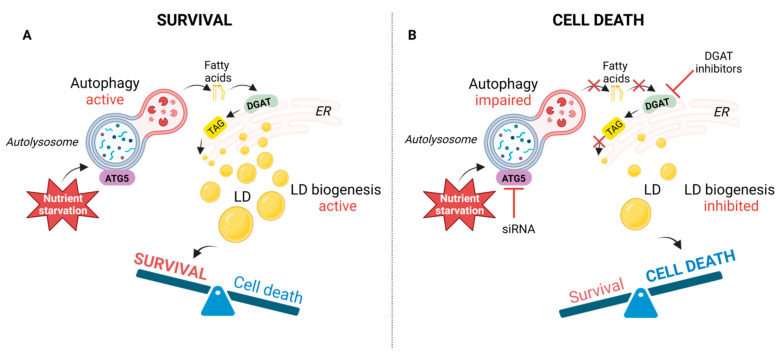
Autophagy-governed and DGAT-mediated LD biogenesis protect HeLa cells from cell death during amino-acid-starvation-induced severe nutrient stress. (**A**) Our data support a model in which HBSS-starvation-induced autophagy promotes LD biogenesis in cancer cells. The biogenesis of LDs under these conditions is dependent on diacylglycerol acyltransferase (DGAT) activity and protects HeLa cells from amino-acid-starvation-induced cell death. (**B**) Blocking autophagy by silencing autophagic gene 5 (ATG5) and LD biogenesis by inhibition of DGAT activity increases HeLa cancer cell death during amino-acid-starvation-induced severe nutrient stress.

This study shows that HeLa- and MDA-MB-231 cancer cells under HBSS starvation conditions accumulate LDs, whose synthesis is governed by upregulation of autophagy (Figure 6). In exploring the role of DGAT enzymes, we demonstrated that inhibition of DGAT1, but not DGAT2, prevents amino-acid-starvation-induced LD accumulation in HeLa cells, suggesting its prerequisite role in the process. Using live cell imaging, we found that ATG5 knockdown HeLa cells lose the capacity for new LD formation during HBSS starvation but contain less LDs with a bigger diameter that remain present in the cells from the cultivation in complete medium. Under fed and mild serum starvation conditions, we found increased LD accumulation in ATG5-silenced HeLa cells and together with reduced LC3 turnover, this suggests that lipophagy might be active and contribute to LD breakdown under these conditions (Figure 6). Importantly, blocking DGAT-mediated LD biogenesis and silencing of ATG5 significantly increased HeLa cell death under severe amino acid starvation conditions. On the other hand, acutely amino acid-starved MDA-MB-231 were less sensitive to the inhibition of autophagy and DGAT activity. As discussed below, our findings support the results of recent studies in MEFs [9,28,45] that describe the broader question of how cells adapt their FA flow and storage during mild and severe nutrient stress to maintain mitochondrial function and cell viability. Our data imply the importance of LD biogenesis and autophagy for HeLa cell survival during severe amino acid starvation conditions and suggest the distinct sensitivity of different cancer cells to the inhibition of both processes.

Although other studies have already shown increased LD synthesis upon amino acid and serum starvation in different cell types, including HeLa cells [9,25,28], the requirement of bulk autophagy as a source of FAs that govern LD biogenesis during severe nutrient stress has been demonstrated only in MEFs [9,28]. In the absence of exogenous lipoproteins and serum, the synthesis of TAG required for LD synthesis depends on pre-existing sources of FAs built in phospholipid membranes and organelles. Mobilization of phospholipid-linked FAs for LD biogenesis induced by stress has been found to be regulated by distinct processes, including intracellular phospholipase A2 VIA-mediated FA release from phospholipid hydrolysis and bulk autophagy-mediated phospholipid recycling [28,46]. In MEFs, amino-acid-starvation-induced LD accumulation was prevented by pharmacological inhibition of autophagy or silencing of ATG5, which reduced the transfer of cellular phospholipids to LDs [28]. Under these conditions, the DGAT1 enzyme mediates FA channeling into LDs downstream of mTOR1-regulated bulk autophagy and protects against lipotoxic disruption of mitochondrial function and promotes cell survival [9]. As an extension of these studies, we sought to investigate how different starvation conditions affect LD dynamics and autophagy in HeLa cells. We demonstrate that mild serum starvation conditions increase LD breakdown, while depletion of both serum and amino acid stimulates LD accumulation during acute starvation (Figure 1, Supplementary Figure S1). These results support findings that LDs are primarily broken down during mild starvation conditions such as serum deprivation, while starving cells in HBSS medium or complete nutrient deprivation promotes LD synthesis [9,25,27,28,47]. Treating cells with DGAT1 and DGAT2 inhibitors revealed that amino-acid-starvation-induced LD synthesis primarily depends on DGAT1 activity (Figure 1), which has also been reported by others [9]. DGAT enzymes catalyze the same reaction and account for nearly all TG synthesis in the cell. Although they can have distinct functions in various conditions that promote fat storage, DGAT1 and DGAT2 can easily compensate for each other, as recently reported [48].

Nutrient deprivation induces autophagy, which enables recycling of cellular components via lysosomal degradation to provide nutrients and building blocks for essential cellular processes during nutrient- and other types of stress [30]. To determine the activation of autophagy, we tracked LC3 turnover and autophagosome levels. Our findings suggest that a detectable level of autophagic flux is present even in cells cultivated in a complete medium, and that this flux is further stimulated by nutrient depletion. This effect is modest upon serum starvation conditions but becomes more evident during HBSS starvation conditions. Although pharmacological inhibition of autophagy suppressed amino-acid-starvation-induced LD accumulation in HeLa- and MDA-MB-231 cells (Figure 2), silencing of ATG5 did not completely reduce neutral lipid content in these cells (Figure 3). Since the silencing was performed before the cells were starved, we hypothesized that LDs could remain present in the cells from the cultivation in complete media. We performed time lapse experiments tracking LD synthesis within several hours of starvation and performed pulse chase assay using two FA analogues that enabled us to distinguish between LDs formed during feeding in complete medium following LD breakdown in serum-depleted medium, and de novo-formed LDs when cells are switched to HBSS starvation [28,44]. Our results indicate that ATG5 silencing prevents LD breakdown that occurs in a complete medium and serum pre-starvation but blocks the formation of new LDs upon HBSS starvation (Figure 4). This suggests that ATG5-mediated autophagy is involved in LD dynamics in HeLa cells, contributing to lipophagy under fed and milder serum starvation conditions and to LD synthesis during severe nutrient stress induced by amino acid depletion.

Cancer cell survival during stress depends on the availability of FAs that are either synthesized de novo within cells, recycled from pre-existing sources of lipids, such as TAGs and phospholipids, or taken up from the extracellular environment [3,4,14,49]. Blocking these pathways may reveal their relevance for cancer cell survival under stressful conditions. Our results suggest that blocking autophagy and DGAT-mediated LD synthesis significantly increases HeLa cancer cell death during acute amino acid starvation, while starved MDA-MB-231 cells are less sensitive to the inhibition of both processes. This is not surprising, since cancer cells vary in their dependence on autophagy and lipid metabolism, which may both be controlled to various extents by contributions from oncogenic drivers and context-dependent cellular states [50,51,52,53]. In our experimental conditions, the cell death of the Ras-driven MDA-MB-231 cells was not significantly affected by the impairment of autophagy or LD biogenesis or a combination of both. The Ras oncogene has been shown to support the survival of various types of cancer cells under various conditions of severe nutrient and oxidative stress [5]. On the other hand, the sensitivity of HeLa cells to the inhibition of autophagy has been associated with the Janus kinase/signal transducer and activator of the transcription 3 (JAK/STAT3) pathway [51], which is strongly activated in starving HeLa cells [54].

The role of DGAT-mediated LD synthesis in mitigating FA lipotoxicity has been recognized in cells exposed to different metabolic stresses, including increased autophagic flux in severely starved cells [9,23,24,55]. In severely starved MEFs and cancer cell lines, the synthesis of TAGs is a major source of FAs for mitochondrial energy production [25], but TAG synthesis also prevents FA-induced mitochondrial damage [9,28]. Our results suggest that severely amino acid-starved HeLa- and MDA-MB-231 cancer cells have different capacities to mitigate the lipotoxicity of free FAs released upon the inhibition of TAG synthesis, which is important for their survival during starvation (Figure 5). Re-esterification of FAs into TAGs not only protects from lipotoxicity, but also regulates the oxidation of FAs and controls the cellular energy state [56]. In MEFs, the transfer of FAs from autophagy-derived LDs to mitochondria is regulated by the main cytosolic lipase ATGL [9,28]. We have shown previously that ATGL-mediated TAG lipolysis is not necessary for MDA-MB-231 cell survival during prolonged serum deprivation [23]. The results of this study suggest that the silencing of ATGL contributes to increased LD accumulation, but it does not significantly affect HeLa cancer cell death during acute serum or amino acid starvation (Figure 4). It would be interesting to determine whether other lipases may compensate for the inhibition of ATGL-mediated lipolysis for FA transfer and contribute to cell survival under these conditions. Taken together, targeting LDs and autophagy could be a valid strategy in the fight against the resistance of some cancer cell types to nutrient stress. In this regard, further studies are required to determine the dependence of cancer cells with distinct genetic backgrounds on the LD/autophagy axis. Finally, given the complex relationship between LDs and autophagy/lipophagy, it will be essential to understand the underlying molecular mechanisms that define the context-dependent roles of autophagy/lipophagy in the regulation of LD metabolism.

## 5. Conclusions

Understanding how cancer cells adapt to stressful stimuli is essential for compromising tumor growth and metastasis. The results of this study demonstrate that LD formation occurs in a DGAT1- and autophagy-dependent manner during amino acid starvation in HeLa- and MDA-MB-231 cancer cells. However, whereas the inhibition of autophagy and LD biogenesis does not increase MDA-MB-231 cell death in these conditions, it is lethal to severely starved HeLa cells. The results of this study highlight the necessity for additional investigations into the intricate interplay between LD metabolism, autophagy, and other cellular processes that contribute to the resilience of cancer cells to nutrient stress.

## Data Availability

Data is contained within the article and Supplementary Material.

## Supplementary Materials

The following supporting information can be downloaded at: https://www.mdpi.com/article/10.3390/cancers15194857/s1, Figure S1: Amino acid starvation induces LD accumulation in HeLa cancer cells; Figure S2: Densitometric analyses of Western blots in ATG5-deficient and control siRNA-treated cells in the presence or absence of autophagy modulators.

## Abbreviations

AA, amino acid; ATG5, autophagy-related protein 5; ATGL, adipose triglyceride lipase; ATP, adenosine triphosphate; BafA1, bafilomycin A1; CE, cholesterol ester; CQ, chloroquine; DGAT, diacylglycerol acyltransferase; FA, fatty acid; LC3, microtubule-associated protein light chain 3; LD, lipid droplet; MEF, mouse embryonic fibroblast; mTORC1, mammalian target of rapamycin complex 1; TAG, triacylglycerol.
