# Supplementary material for: The Combined Inhibition of Autophagy and Diacylglycerol Acyltransferase-Mediated Lipid Droplet Biogenesis Induces Cancer Cell Death during Acute Amino Acid Starvation
Source: Cancers (Basel). 2023 Oct 5;15(19):4857. doi: 10.3390/cancers15194857 (PMC10571868; doi:10.3390/cancers15194857)
Supplement: Supplementary file 1 [file cancers-15-04857-s001.zip › cancers-2468725-supplementary.pdf]

Supplementary Material

Article

The Combined Inhibition of Autophagy and Diacylglycerol Acyltransferase-Mediated Lipid Droplet Biogenesis Induces Cancer Cell Death during Acute Amino Acid Starvation

by Maida Jusović, Pia Starič, Eva Jarc Jovičić and Toni Petan

Contents:

Supplementary Figures and Figure Legends

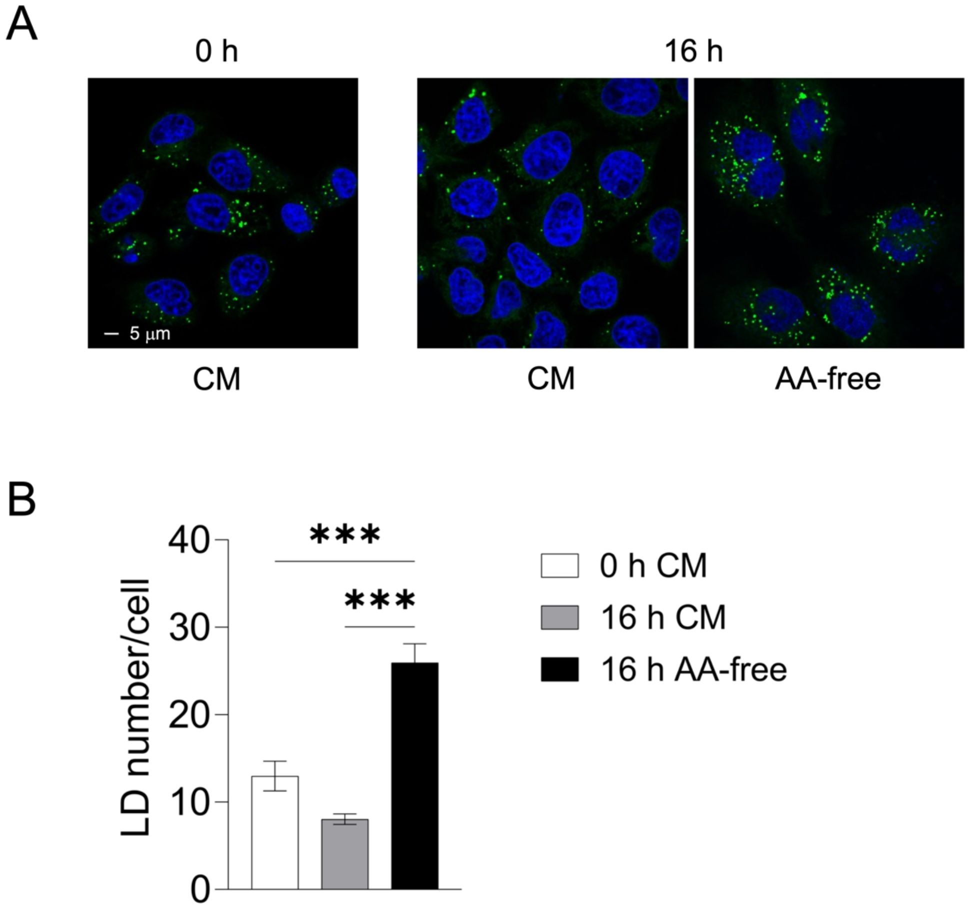

Suppl. Figure S1. **Amino acid starvation induces LD accumulation in HeLa cancer cells.** (A, B) Microscopic images and quantitative analysis of LD abundance in fed and starved HeLa cells. HeLa cells were grown for 24 h in complete medium (CM; 0 h) and then the medium was replaced with fresh CM or with amino acid (AA)-free HBSS buffer for the next 16 h. (A) LDs and nuclei were visualised using 1 µg/ml BODIPY 493/503 and Hoechst stain solution, respectively. (B) Images from confocal microscopy were analysed using ImageJ and the LD Counter Plugin for LD quantification. The data presented are means ± SEM obtained on n > 50 cells/sample. Statistically significant differences are indicated (*, $P < 0.05$; **, $P < 0.01$; ***, $P < 0.001$; one-way ANOVA with Šidak adjustment).

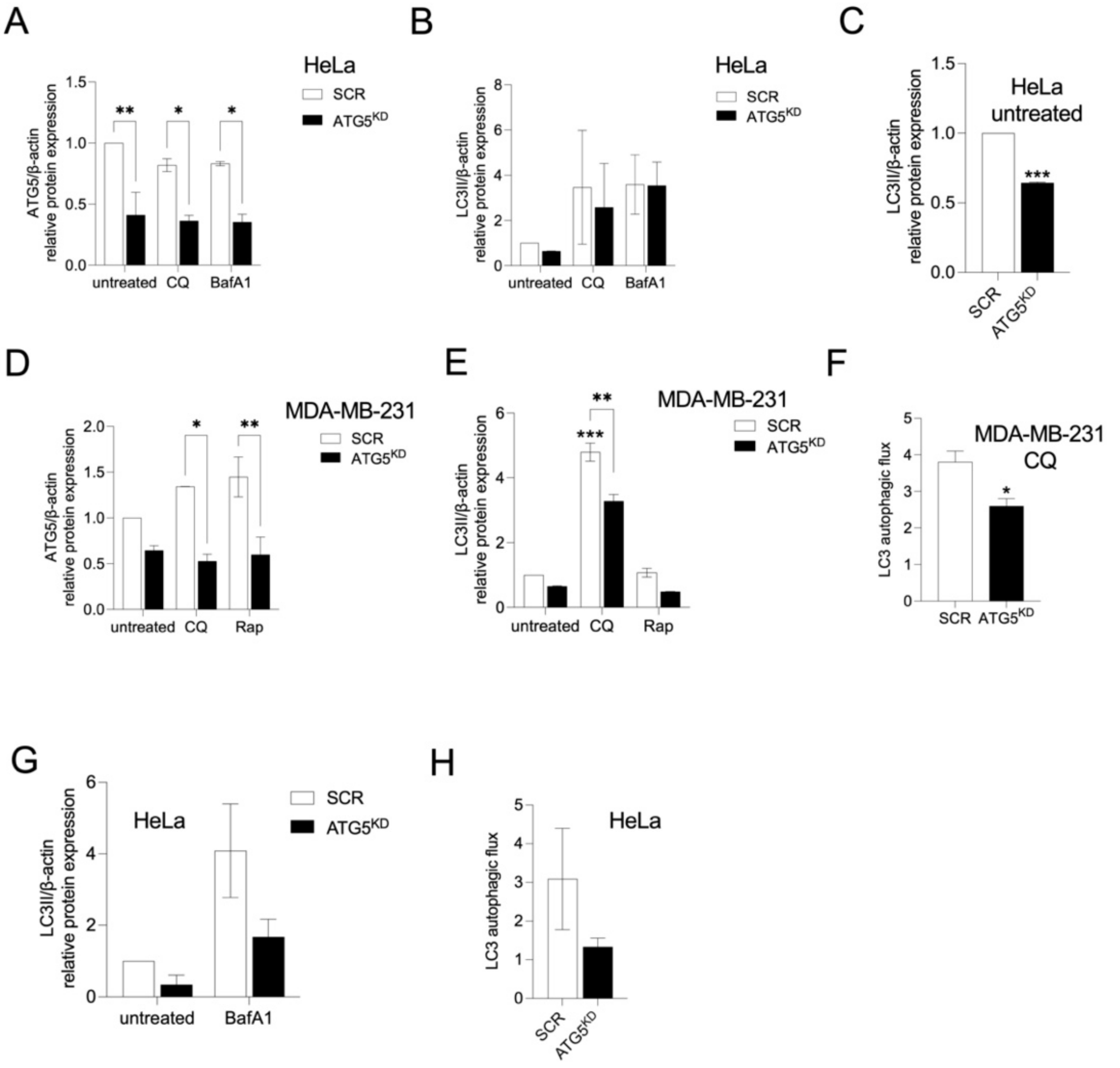

Supp. Figure S2. Densitometric analyses of western blots in ATG5-deficient and control siRNA-treated cells in the presence or absence of autophagy modulators. (A–C) Densitometric analysis of protein levels of ATG5 and LC3-II corresponding to experiment shown in Fig. 3E. (D–F) Densitometric analysis of protein levels of ATG5 and LC3-II and autophagic flux correponding to experiment shown in Fig. 3F. (G, H) Densitometric analysis of LC3-II protein levels and autophagic flux correponding to experiment shown in Fig. 4B. (F, H) Autophagic flux was determined in SCR and ATG5 KD cells by calculating the difference between CQ-treated (F) or BafA1-treated (H) samples and corresponding vehicle control samples. Data are means ±SEM of two independent experiments. Results that are statistically significant are indicated (*, $P < 0.05$; **, $P < 0.01$; ***, $P < 0.001$ (two-way ANOVA with Šidák adjustment (A, E, F), unpaired t-test (G)). BafA1, bafilomycin A1; CQ, chloroquine; Rap, rapamycin.
